# Expeller-Pressed Canola (*Brassica napus*) Meal Modulates the Structure and Function of the Cecal Microbiota, and Alters the Metabolome of the Pancreas, Liver, and Breast Muscle of Broiler Chickens

**DOI:** 10.3390/ani11020577

**Published:** 2021-02-23

**Authors:** G. Douglas Inglis, Benjamin D. Wright, Stephanie A. Sheppard, D. Wade Abbott, Matt A. Oryschak, Tony Montina

**Affiliations:** 1 Lethbridge Research and Development Centre, Agriculture and Agri-Food Canada, Lethbridge, AB T1J 4B1, Canada; wade.abbott@canada.ca; 2 Department of Biological Sciences, University of Lethbridge, Lethbridge, AB T1K 3M4, Canada; wrightb@uleth.ca (B.D.W.); stephanie.sheppard@uleth.ca (S.A.S.); 3 Department of Chemistry and Biochemistry, University of Lethbridge, Lethbridge, AB T1K 3M4, Canada; 4 Alberta Agriculture and Forestry, Edmonton, AB T6H 5T6, Canada; oryschak@ualberta.ca; 5 Southern Alberta Genome Sciences Centre, University of Lethbridge, Lethbridge, AB T1K 3M4, Canada

**Keywords:** broiler chickens, canola meal, cecum, microbiota, pancreas, liver, breast muscle, metabolome

## Abstract

**Simple Summary:**

The impact of a broiler chicken diet that is supplemented with canola meal (CM) on the cecal microbiota, growth, and metabolome of broiler chickens was examined. CM did not affect feed consumption, weight gain, nor the richness, evenness, or diversity of the cecal bacterial community. However, CM affected the structure of the bacterial microbiota, the concentrations of short-chain fatty acids (SCFAs) were elevated, and the abundance of bacterial taxa known to ferment dietary fiber were more abundant in the ceca of birds fed the CM diet. Nuclear magnetic resonance spectroscopy metabolomics was applied, and a number of metabolites that were associated with SCFA metabolism were differentially regulated in cecal digesta. The supplementation of diet with CM also affected the metabolic profiles of the pancreas, liver, and breast muscle (e.g., metabolites that are associated with energy production, protection against oxidative stress, and pathways of amino acid and glycerophospholipid metabolism). In summary, broiler chickens that were fed a diet supplemented with CM showed equivalent feed consumption and growth, but CM affected the composition and function of the cecal microbiota, and the metabolome of the pancreas, liver, and breast muscle of birds indicating the possibility of an increased disease risk. The study also demonstrated the utility of using metabolomics to ascertain impacts of diet on the microbiota and host tissues, and identified biomarkers to facilitate subsequent evaluation in production settings.

**Abstract:**

The inoculation of one-day-old broiler chicks with the cecal contents from a mature broiler breeder resulted in a highly diverse and uniform cecal bacterial community. CM did not affect feed consumption, weight gain, nor the richness, evenness, or diversity of the cecal bacterial community. However, the structure of the bacterial community was altered in birds fed the CM diet. Although the CM diet was formulated to contain equivalent metabolizable energy to the control diet, it contained more dietary fiber. The abundance of bacterial families, including those that are known to contain species able to metabolize fiber was altered (e.g., bacteria within the families, *Methanobacteriaceae*, *Atopobiaceae*, *Prevotellaceae*, *Clostridiales* Family XIII, *Peptostreptococcaceae*, and *Succinivibrionaceae*), and concentrations of SCFAs were higher in the ceca of birds fed the CM diet. Moreover, concentrations of isoleucine, isobutyrate, glutamate, and 2-oxoglutarate were higher, whereas concentrations of phenyllactic acid, indole, glucose, 3-phenylpropionate, and 2-oxobutyrate were lower in the digesta of chickens that were fed CM. The metabolic profiles of pancreas, liver, and breast muscle tissues of birds fed the CM diet differed from control birds. Metabolites that were associated with energy production, protection against oxidative stress, and pathways of amino acid and glycerophospholipid metabolism had altered concentrations in these tissues. Some of the observed changes in metabolite levels may indicate an increased disease risk in birds fed the CM diet (e.g., pancreatitis), and others suggested that birds mounted metabolic response to offset the adverse impacts of CM (e.g., oxidative stress in the liver).

## 1. Introduction

Canola (*Brassica napus*) is a major annual field crop grown in North America, with >5.0 million ha in production annually in Canada [1]. Canola meal (CM), the protein rich by-product generated following oil extraction from canola seeds, has shown promise in feeding applications in monogastric species when applied at a low inclusion rate [2]. Thus, CM represents a potential value-added product for canola residues [3]. A number of studies have examined the impact of rapeseed meal (RM) and, to a lesser extent, CM on the health and performance of layer and broiler chickens. Historically, the use of CM in poultry feedstuffs has been limited due to its correlation with weaker performance outcomes when compared to soybean meal, despite containing similar levels of protein (37% in CM and 46% in soybean meal) [4]. This outcome has been attributed to the higher abundance of plant cell wall polysaccharides (32% in CM and 22% in soybean meal), lignin (10% in CM and 3% in soybean meal) in CM [5,6], and as a result of the poor digestibility of CM/RM proteins as compared with soybean proteins [7]. 

Relatively few studies have examined the impact of CM or RM on the composition and function of the enteric microbiota in chickens. In this regard, Long et al. [8] examined the impacts of RM on the cecal microbiota of laying hens at 50 weeks-of-age, and Toghyani et al. [9] and Konieczka et al. [10] examined the impact of CM/RM supplementation of diets on the cecal microbiota of broilers, but they did not apply next-generation sequence technologies, nor did they examine bacterial function. As the impacts of CM on the cecal microbiota and function remains largely unexplored, a primary goal of the current study was to comparatively examine the impacts of a diet that was supplemented with CM relative to a control diet on broiler performance, and the structure and function of the cecal microbiota using both next generation sequencing and a nuclear magnetic resonance spectroscopy (NMR)-based metabolomics approach. Metabolomics is the quantitative study of small molecules (metabolites) that are involved in various biochemical processes and it provides a functional measure of downstream effects that result from both internal and external stimuli, including diet; however, metabolomics has not been extensively applied to elucidate functional processes and dietary responses in chickens. To better ascertain the impacts of dietary CM on broilers, the metabolomic profile of cecal digesta, as well as pancreas, liver, and breast muscle tissues, was examined. These three tissues have an important role in carbohydrate metabolism [11], digestion [12], and in the health status of birds [13], respectively; in addition, breast tissue is a high value meat outcome of broiler production. Finally, the metabolome of the cecal digesta provides a key insight into the functioning of the microbiota present in the cecum and reflects changes in community structure observed using sequencing.

We hypothesized that the inclusion of CM in broiler diets would affect bird performance in a controlled setting and would, correspondingly, affect the structure and function of the cecal microbiota in relation to polysaccharide fermentation, and affect the metabolome of pancreas, liver, and breast muscle. To test this hypothesis, the objectives of the study were to: formulate diet treatments that were equivalent in metabolizable energy (±CM); establish a uniform microbiota that is representative of broilers in a production setting; measure feed efficiency in broilers over a 35-day period; apply Illumina technology and microbiota bioinformatics pipelines to ascertain the structure of the cecal microbiota; measure SCFAs in cecal digesta; and, apply proton NMR-based metabolomics in order to comparatively examine the metabolome within cecal digesta, pancreas, liver, and breast muscle.

## 2. Materials and Methods

### 2.1. Ethics Statement

The study was carried out in strict accordance with the recommendations that were established in the Canadian Council on Animal Care Guidelines. The project was reviewed and approved by the Agriculture and Agri-Food Canada (AAFC) Lethbridge Research and Development Centre (LeRDC) Animal Care Committee (Animal Use Protocol Review #1810).

### 2.2. Experimental Design and Diet Treatments

The experiment was arranged as a completely randomized design with two levels of diet (±CM supplementation) and six levels of replicate. The starter (10 days), grower (14 days), and finisher (11 days) control diets used were corn-based formulations that are commercially used in Canada (Table 1). For the CM diet, the basal diet was supplemented with expeller-pressed CM (involves the physical separation of oil and meal without heat; <60 °C) from the Hartland Colony, Bashaw, AB, Canada (https://www.pleasantvalleyoilmills.com/ (accessed on 17 February 2021)) at a concentration of 20% (*w*/*w*) using the same starter, grower, and finisher schedule as the control diet. The canola cultivar used to generate the CM was not genetically modified (e.g., Roundup Ready^®^ or LibertyLink^®^). Care was taken to ensure that the two diets were energetically equivalent (Table 2). All of the diet ingredients were sourced locally, and both diets were formulated at the AAFC LeRDC Feed Mill, and chemical analyses of the diets were completed, as described in Oryschak et al. [14].

### 2.3. Broilers

One-day-old broiler chicks (Ross 308) were obtained from the Sunrise Hatchery (Lethbridge, AB, Canada) in two groups of 30 that were hatched six days apart; the two groups of birds that were obtained on separate occasions comprised different replicates and they were treated identically with respect to the experimental design (e.g., the assignment of treatments and time course of data collection). All of the chicks were hatched from eggs that were sourced from the same broiler breeder farm. Chicks were transported from the hatchery to the Livestock Containment Unit (LCU) at LeRDC. Upon arrival at the facility, each chick was identified with a numbered ankle bracelet, and they were weighed. The ambient temperature within the LCU animal rooms was set to 30 °C. From each hatch, 15 randomly selected chicks were placed in one custom stainless steel elevated enclosure (Alternative Design Manufacturing and Supply, Inc., Siloam Springs, AR, USA), and the remaining 15 chicks were placed in another identical enclosure. The enclosures were arranged so that there was no possibility of contact between birds in adjacent enclosures. The enclosures were sanitized before placement of the chicks, and brown kraft postal paper was placed on the slotted floor of the enclosures. Each cage was equipped with a 30 × 30 cm brooder (Titan Incubators, Malmesbury, UK), a 24″ chick feeder (United Farmers of Alberta Co-operative Ltd., Lethbridge, AB, Canada), and a Bell waterer (United Farmers of Alberta Co-operative Ltd.). Chicks in one of the enclosures were provided a control starter diet, and chicks in the remaining enclosure were provided a starter diet that was supplemented with CM.

### 2.4. Cecal Content Collection for Chick Inoculation

All of the chicks were administered cecal contents that were harvested from healthy mature broiler breeder male donors (≈30 weeks-of-age). The donors were humanely euthanized on farm using a compressed CO_2_ captive bolt device (Jarvis Industries Canada Ltd., Calgary, AB, Canada), and immediately transferred to LeRDC. Individual birds were placed into a sanitized plastic container, and the feathers were removed. The skin was sanitized with Peroxigard™ (Virox Technologies Inc., Oakville, ON, Canada), a laparotomy was conducted, and the ceca were ligated using sterile cable ties (CT6BK30-C; 3M, St. Paul, MN, USA) to prevent infiltration of air. The ceca were incised and transferred into a Thermo Forma 1025 anaerobic chamber (ThermoFisher Scientific Inc., Waltham, MA, USA) containing a 85% N_2_: 10% CO_2_: 5% H_2_ atmosphere. Within the chamber, ceca were placed on a sterile large petri-dish (20-cm-diameter), the cable ties were removed, and, using a sterile wooden splint, the cecal contents were pushed into a sterile pre-weighed 500 mL Pyrex™ medium bottle (Fisher Scientific, Ottawa, ON, Canada). The digesta was suspended in reduced phosphate buffered saline (PBS) at a 3:1 ratio (PBS: digesta *v*/*w*), transferred in 1.5 mL aliquots into 2.0 mL screw cap tubes, snap frozen in liquid nitrogen, and then stored at −80 °C until used.

### 2.5. Enteric Microbiota Establishment

Cecal digesta was thawed in an anoxic atmosphere, the slurry was transferred into sterile 3 mL syringes (1 mL per syringe), and the syringes were capped in order to prevent oxygen infiltration and placed in sealed plastic bags (eight syringes per bag). Each plastic bag was placed in a thermos containing a pre-heated (37 °C) ThermoSafe PolarPack (Fisher Scientific), and the syringes were at transported to the LCU. Chicks (two-day-old) were orally inoculated with the cecal digesta slurry within ≈20 min. of removal of the syringes from the anoxic atmosphere. Each syringe was fitted with a flexible disposable PTFE feeding needle (15G 3″ 2.8-mm-diameter ball) (Cadence Inc., Cranston, RI, USA). Individual chicks were gently restrained, the beak was carefully opened by inserting the feeding needle, and the feeding needle was carefully advanced into the mouth and then guided into the esophagus. Once in place, 1 mL of the cecal slurry was delivered into the esophagus, and the needle was carefully removed. Chicks were observed for 30 min., and none of the birds showed any evidence of distress.

### 2.6. Feed Consumption and Weight Gain

The chicks were retained in the two groups for the first five days, at which point five randomly-selected chicks per diet treatment were placed into three new sanitized enclosures containing a brooder, a feeder, and a water. These individual cages represented a replicate with five observations (i.e., birds) per replicate (*n* = six replicates per diet treatment). The floor of the enclosure was papered; however, the flooring under the area around the feeder was left unpapered in order to allow unconsumed feed to fall through the enclosure slots onto plastic trays situated on the animal room floor below the enclosures. Feed that was provided in the morning was weighed using a Pelouze scale (model 4010; Uline, Milton, ON, Canada), and residual feed within the feeder, on the paper within the enclosure, and on the plastic tray below the enclosure at the end of the 24-h period was weighed daily using a Mettler scale (Model BB2440 DeltaRange; Mettler Toledo, Mississauga ON, Canada). Care was taken to ensure that feces was removed from the residual feed. Waterers were filled, changed, and washed as needed. Identification ankle bracelets were regularly checked to ensure that they were not becoming too tight as the birds grew. Bracelets were typically replaced once per week if needed. The birds were weighed at seven-day intervals for 35 days.

### 2.7. Necropsies

When birds reached 35 days-of-age, they were anesthetized within an induction chamber with isoflurane (WDDC, Edmonton, AB, Canada) using an E-Z anesthesia machine (E-Z Systems, Palmer, PA, USA). Once anesthetized, the birds were transferred to an anesthesia mask system that was located within a fume hood. Under anesthesia, blood was intracardially collected, serum was separated using BD Vacutainer^®^ red topped tubes (Becton Dickinson; Mississauga, ON, Canada), and stored at −80 °C until processed. The birds were then humanely euthanized by decapitation (NS-804; Braintree Scientific, Braintree, MA, USA). Feathers on the breast area were removed, the skin was sanitized with Peroxigard™, an incision in the skin was made, and a sample of breast muscle (middle portion of the right breast) was removed for metabolomic analysis. A ventral laparotomy was performed, viscera was exposed, ceca were removed, incised, and cecal contents were carefully removed for the analysis of SCFAs, characterization of bacterial communities by Illumina sequencing, and metabolomics analyses. In addition, the pancreas (lower portion at the open end of the small intestine) and liver (lower left lobe) were removed for metabolomics analysis. Cecal digesta for bacterial community characterization and metabolomics, and inferior pancreas, part of the inferior left lobe of the liver, and breast muscle samples for metabolomics were snap frozen. All of the samples were maintained at −80 °C until they were processed. The sex of all birds was determined at necropsy (i.e., by the presence of testes).

### 2.8. Short-Chain Fatty Acid Analysis

Concentrations of SCFAs were determined, as described previously [15,16]. Briefly, cecal digesta was homogenized in phosphate buffered saline (pH 7.2) at a 1:9 ratio (*w*/*v*). Meta-phosphoric acid (Sigma Aldrich, Oakville, ON, Canada) was added to the homogenate at a 1:4 ratio (*v*/*v*), and then incubated at room temperature for 30 min. The samples were centrifuged at room temperature for 75 min. at 16,100× *g*, and the supernatants were collected and stored at −20 °C. Concentrations of acetate, propionate, isobutyrate, butyrate, valerate, isovalerate, and caproate concentrations were quantified with a gas chromatograph (Agilent Technologies, Model 6890N with 7683 Series Injector; Agilent Technologies Canada Inc., Mississauga, ON, Canada) according to established protocols [17,18]. In addition, concentrations of total SCFAs, as well as the ratio of two carbon versus three carbon SCFAs, were determined.

### 2.9. Characterization of Bacterial Communities

The total DNA from cecal contents was extracted from 200 mg ± 20 mg of cecal digesta using the QIAamp^®^ PowerFecal^®^ Pro DNA kit (Qiagen Inc., Toronto, ON, Canada) according to manufacturer’s protocols. This kit includes a bead beating step, and is designed to effectively extract genomic DNA from both Gram positive and Gram negative bacteria. The extracted DNA was quantified using a Qubit 4 fluorometer (Thermo Fisher Scientific) and PCR amplified for 16S rRNA gene metagenomics analyses using the protocol developed by Kozich et al. [19], which targets the V4 region of the 16S rRNA gene. The PCR mastermix included 12.5 µL of Paq5000 Hi Fidelity Taq Master Mix (Agilent Technologies Canada Inc.), 1 µL of 10 µM forward primer, 1 µL of 10 µM reverse primer (Integrated DNA Technologies, Coralville, IA, USA), 8.5 µL of Nuclease-Free Water (Qiagen Inc.), and 2 µL of genomic DNA template (30–50 ng). The reactions were amplified on an Eppendorf thermocycler (Mastercycler Pro S; Eppendorf, Mississauga, ON, Canada) using the following conditions: 95 °C for 2 min.; 25 cycles of 95 °C for 20 sec, 55 °C for 15 sec, and 72 °C for 2 min.; and, a final elongation cycle at 72 °C for 10 min. Amplicons were purified with AMPure XP beads (Beckman Coulter Diagnostics, Brea, CA, USA), and checked for quality and size with a Bioanalyzer 2100 (Agilent Technologies Canada Inc.). Quantification of the amplicons was done using the Qubit 4. The samples were normalized to 4 nM, pooled, denatured with NaOH, and further diluted with HT1 (Illumina, San Deigo, CA, USA) to produce a 4 pM library for sequence analysis. Twenty percent PhiX control DNA was added to the library as a sequencing control. The library was loaded using a MiSeq Reagent Kit v2 500-cycle, and run on an Illumina MiSeq platform (Illumina). The Q30 score for the output data was 89%. 

Quantitative Insights Into Microbial Ecology 2 (QIIME™ 2, version 2019.10) [20] was used to process and classify bacterial reads. The raw reads were denoised with DADA2 [21], and representative sequences and amplicon sequence variants (ASV) were generated. Low quality reads (quality score <20) and samples with a read depth of less than 22,000 were removed from the analyses. A phylogenetic tree of ASV sequences was generated, and the taxonomy of each ASV was identified using a machine learning classifier that was pre-trained with the reference SILVA 132 database (release version 132; lva-132-99-515-806-nb-classifier.qza) [22]. Core metric analyses were completed in QIIME2 to obtain evenness (Pielou’s), alpha diversity (Faith’s and Shannon’s), and beta diversity (Jaccard’s, Bray–Curtis, and unweighted and weighted UniFrac distances).

### 2.10. Metabolomics

The samples (150 mg) were suspended in metabolomics buffer (0.125 M KH_2_PO_4_, 0.5 M K_2_HPO_4_, 0.00375 M NaN_3_, and 0.375 M KF; pH 7.4). Liver and pancreas tissue as well as cecal digesta were homogenized using a Bullet Blender tissue homogenizer (Next Advance, Troy, NY, USA) with 150 mg of 2-mm-diameter zirconium oxide beads (Next Advance) at setting 8 for 5 min. The breast muscle samples were homogenized with 150 mg of 6-mm-diameter steel beads for 10 min. using a Qiagen TissueLyser LT at 50 Hz (Qiagen Inc.). All of the samples were then centrifuged at 14,000× *g* for 5 min. Each supernatant was passed through a 3000 MWCO Amicon Ultra-0.5 filter (Millipore Sigma, Oakville, ON, Canada) that had been rinsed with Millipore water 10 times immediately prior to use [23]. The filters were then centrifuged at 14,000× *g* for 30 min. at 4 °C. From each sample, 360 μL of the filtrate was mixed with 140 μL of deuterium oxide containing 0.05% *v*/*v* trimethylsilylpropanoic acid (TSP) and 200 μL of metabolomics buffer (final total volume of 700 μL); TSP was used as a chemical shift reference for ^1^H-NMR spectroscopy. The solution was vortexed and then centrifuged at 12,000× *g* for 5 min. at 4 °C to pellet any particulate matter. Following centrifugation, a 550 μL aliquot of the supernatant was loaded in a 5 mm NMR tube, and then run on a 700 MHz Bruker Avance III HD spectrometer (Bruker, Milton, ON, Canada) for spectral collection.

For data acquisition, the Bruker 1-D NOESY gradient water suppression pulse sequence ‘noesygppr1d’ was used with a 10 ms mixing time. Each sample was run for 128 scans to a total acquisition size of 128 k, a spectral window of 20.5 ppm, a transmitter offset of 4.7 ppm, and a recycle delay of 4 sec. All of the measurements were recorded using a Bruker triple resonance TBO-Z probe. The Bruker automation program “pulsecal” was used on each sample before data acquisition to guarantee that the 90-degree pulse was correctly calibrated, ensuring quantitative and comparable data across samples [24]. The spectra were zero-filled to 256k, automatically phased, baseline corrected, and then line-broadened by 0.3 Hz [25]. The spectra were then exported to MATLAB (MathWorks, Natick, MA, USA) as Ascii files, where they underwent Dynamic Adaptive Binning [26] followed by manual inspection and correction. The dataset was then normalized to the total metabolome, excluding the region containing the water peak, and pareto scaled.

### 2.11. Statistical Analyses

The experiment was analyzed as a complete randomized design with two levels of treatment and two levels of bird gender. Replicates that were conducted on separate occasions (i.e., runs) were treated as a random effect. The mixed procedure of SAS (SAS Institute Inc., Cary, NC, USA) was used to analyze feed consumption and weight gain. Before analysis, the normality of variance was confirmed. Variables that were not independent (e.g., feed consumption and weight gain over time) were treated as a repeated measure; the appropriate covariance structure was utilized according to the lowest Akaike’s Information Criterion. In the event of a significant main effect, the least squares means (lsmeans) test was used to compare the treatments within factors. 

Alpha diversity of the cecal microbiota was analyzed by pairwise comparisons of Kruskal–Wallis test. The beta diversity of the microbiota (males versus females, and control versus CM diet) was analyzed by pairwise permutational multivariate analysis of variance (PERMANOVA) [27]. A Benjamini and Hochberg correction was applied to pairwise alpha and beta diversity tests. A table of ASVs was exported from QIIME2 and used to generate compositional barplots and heatmaps in SigmaPlot (Systat, San Jose, CA, USA; version 12.0). The percent abundance of relevant taxa was normalized, and two-way ANOVA using SAS was applied. The lsmeans test was applied in the event of a significant main effect (*p* ≤ 0.05).

Metabolomics analysis was performed using MATLAB (Math Works) and the Metaboanalyst R package [28]. Spectral bins were subjected to univariate analysis in MATLAB in order to determine which metabolites were significantly altered between treatments. The univariate measures were calculated using a decision tree algorithm, as described by Goodpaster et al. [29]. All of the p-values obtained from this analysis were Bonferroni–Holm corrected for multiple comparisons. MATLAB was also used to calculate the percent difference of the bins between treatments. Metaboanalyst R was used to carry out principle component analysis (PCA) and orthogonal partial least squares discriminant analysis (OPLS-DA). Metabolites were identified using Chenomx 8.2 NMR Suite (Chenomx Inc., Edmonton, AB, Canada).

## 3. Results

### 3.1. Supplementation of Diets with Canola Meal Did Not Affect Feed Consumption or Broiler Weight Gain

**Figure 1:**
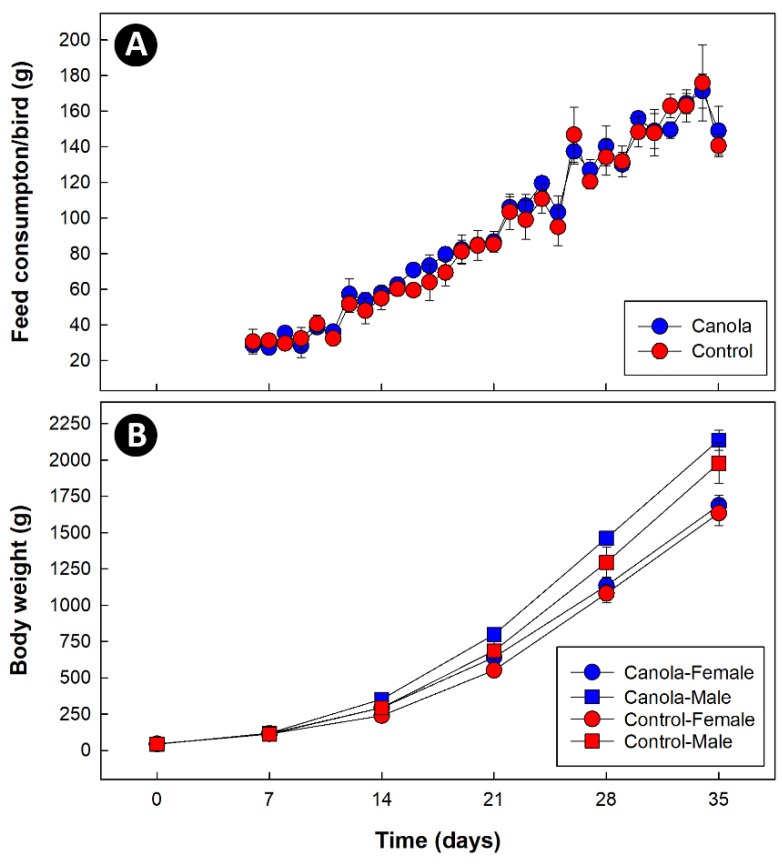
(**A**) Temporal feed consumption (g/bird/d) of broilers fed a diet supplemented with canola meal (20%) relative to a control diet. Feed consumption increased (*p* < 0.001) over time, but there was no difference (*p* = 0.514) between the diet treatments. (**B**) Temporal change in body weights (g) of female and male broilers fed a diet with canola meal (20%) relative to a control diet. Increases in the body weights of female and male birds differed over time (*p* < 0.001), and male birds were heavier (*p* ≤ 0.008) than female birds at day 21. There was no effect (*p* = 0.118) of the diet treatment on body weights.

The rate of feed consumption increased (*p* < 0.001) over time, but there was no difference (*p* = 0.514) between the diet treatments (Figure 1A). Increases in the body weights of the female (*n* = 28) and male (*n* = 26) birds differed over time (*p* < 0.001), and male birds were heavier (*p* ≤ 0.008) than female birds at day 21 and thereafter (Figure 1B). There was no effect (*p* = 0.118) of the diet treatment on body weights.

### 3.2. The Canola Meal Diet Did Not Affect the Richness, Evenness, or Diversity of Cecal Bacterial Communities

The inoculation of two-day-old broiler chicks with the cecal contents from a mature broiler breeder resulted in a highly diverse and uniform cecal bacterial community within and amongst the birds at 35-days-of-age. Bacterial richness, evenness, and diversity of bacterial communities did not differ (*p* ≥ 0.161) between the sexes and diet treatments (Figures S1 and S2).

### 3.3. The Canola Meal Diet Affected the Structure of the Cecal Bacterial Community

**Figure 2:**
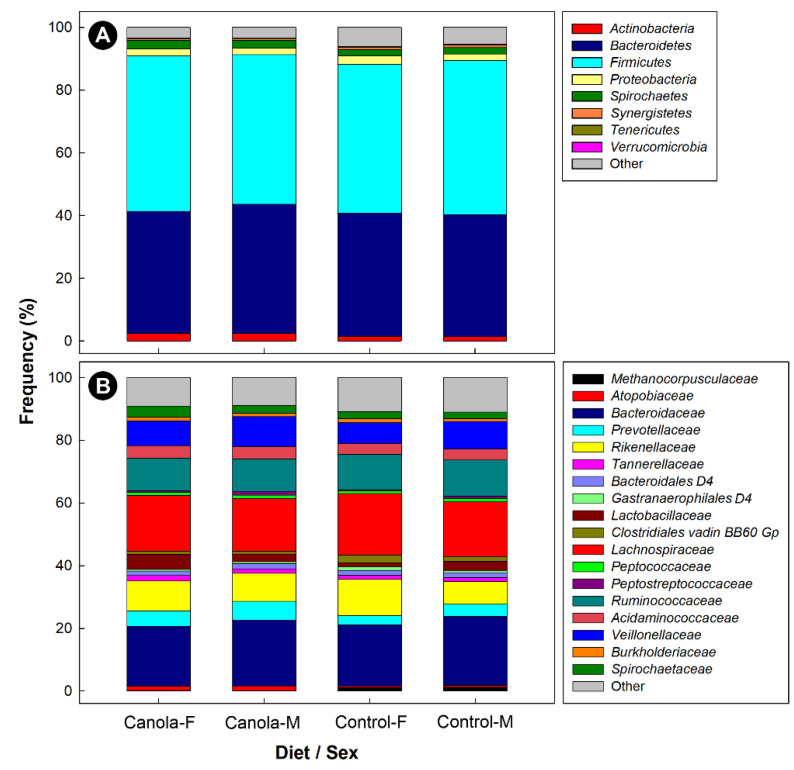
The relative abundance of archaea and bacterial phyla (**A**) and families (**B**) observed in the cecal digesta of 35-day-old female and male broilers fed a diet supplemented with canola meal (20%) or a control diet with ≥22,000 reads (*n* = 43). Data were averaged for female and male birds by diet treatment.

**Figure 3:**
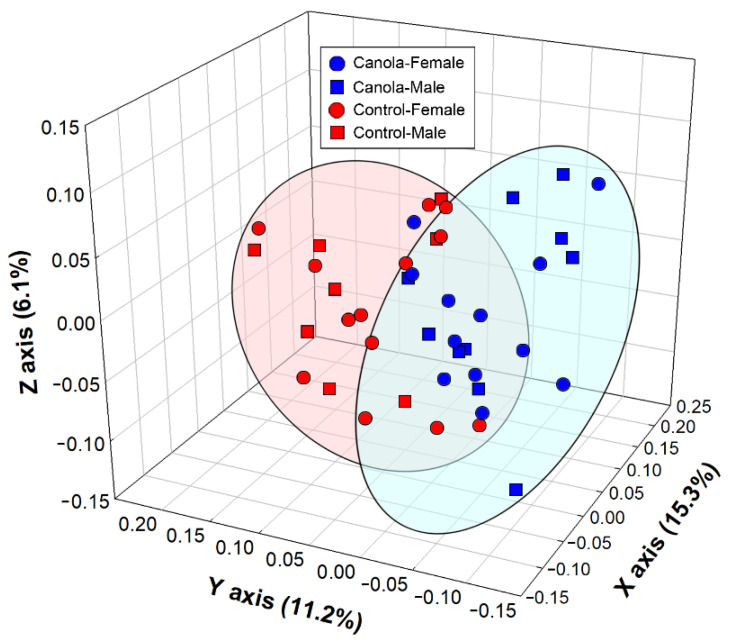
Principle coordinate analysis (unweighted UniFrac) of bacterial communities in the cecal digesta of female and male 35-day-old broilers fed a diet supplemented with canola meal (20%) or a control diet. Ellipsoids highlight the differences in community structure between communities in the digesta of birds fed the canola meal and control diets. The cecal communities differed between the two diets as determined by Jaccard (*p* = 0.001), Bray Curtis (*p* = 0.001), and unweighted (*p* = 0.001) and weighted (*p* = 0.016) UniFrac analyses.

**Figure 4:**
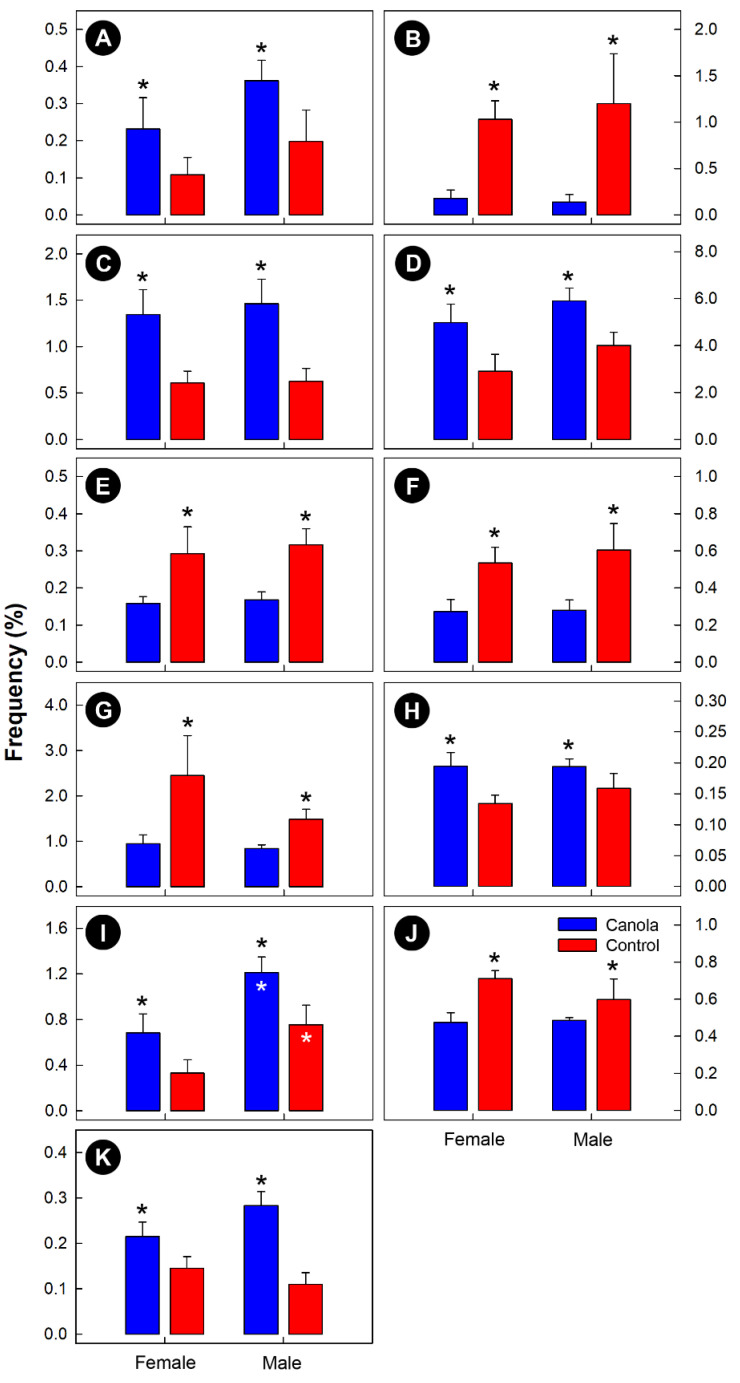
The relative abundance of select archaea and bacterial families (*phylum*-*family* names presented) in the cecal digesta of 35-day-old female and male broilers fed a diet supplemented with canola meal (20%) or a control diet. (**A**) *Archaea-Methanobacteriaceae*. (**B**) *Archaea-Methanocorpusculaceae*. (**C**) *Actinobacteria-Atopobiaceae*. (**D**) *Bacteroidetes-Prevotellaceae*. (**E**) *Bacteroidetes*-*Bacteroidales* D4 uncultured. (**F**) *Firmicutes-Christensenellaceae*. (**G**) *Firmicutes-Clostridiales* vadin BB60 group. (**H**) *Firmicutes-Clostridiales* D4 Family XIII. (**I**) *Firmicutes-Peptostreptococcaceae*. (**J**) *Proteobacteria-Desulfovibrionaceae*. (**K**) *Proteobacteria-Succinivibrionaceae*. The first name is the phylum and second name the family. Vertical lines that are associated with histogram bars are standard error of the means, and histogram bars indicated with a black asterisk indicate that abundances differed (*p* ≤ 0.047) between the two diet treatments. Bars marked with white asterisks indicate that abundances differed (*p* ≤ 0.050) between male and female broilers.

The composition of the microbiota was dominated by bacteria within the *Firmicutes* and *Bacteroidetes*, regardless of sex and diet treatment (Figure 2A). Within the *Firmicutes* phylum, bacteria belonging to the *Lactobacillaceae*, *Lachnospiraceae*, *Ruminococcaceae*, *Acidaminococcaceae*, and *Veillonellaceae* were prominent (Figure 2B). Within the *Bacteroidetes* phylum, bacteria that were assigned to the *Bacteroidaceae*, *Prevotellaceae*, and *Rikenellaceae* predominated. In addition, bacteria within the *Spirochaetes* phylum and *Spirochaetaceae* family were commonly observed. Unweighted UniFrac (*p* = 0.001), weighted UniFrac (*p* = 0.021), Jaccard (*p* = 0.001), and Bray Curtis (*p* = 0.001) analysis indicated that the structure of the cecal microbiota of broilers that were fed the CM diet differed from that of the control birds (Figure 3). A quantitative comparison of taxa (*phylum*-*family*) abundance showed that *Archaea-Methanobacteriaceae*, *Actinobacteria-Atopobiaceae*, *Bacteroidetes-Prevotellaceae*, *Firmicutes-Clostridiales* D4 Family XIII, *Firmicutes-Peptostreptococcaceae*, and *Proteobacteria-Succinivibrionaceae* were more abundant (*p* ≤ 0.047), and *Archaea- Methanocorpusculaceae*, *Bacteroidetes*-D4 uncultured, *Firmicutes-Christensenellaceae*, *Firmicutes-Clostridiales* vadin BB60 group, and *Proteobacteria-Desulfovibrionaceae* were less abundant (*p* ≤ 0.041) in the cecal contents of broilers that were fed the CM-supplemented diet (Figure 4). With the exception of *Firmicutes-Peptostreptococcaceae* (*p* = 0.005), there were no differences (*p* ≥ 0.118) between males and females.

### 3.4. The Canola Meal Diet Contained More Dietary Fiber, and Resulted in Increased Levels of Cecal Fermentation

**Figure 5:**
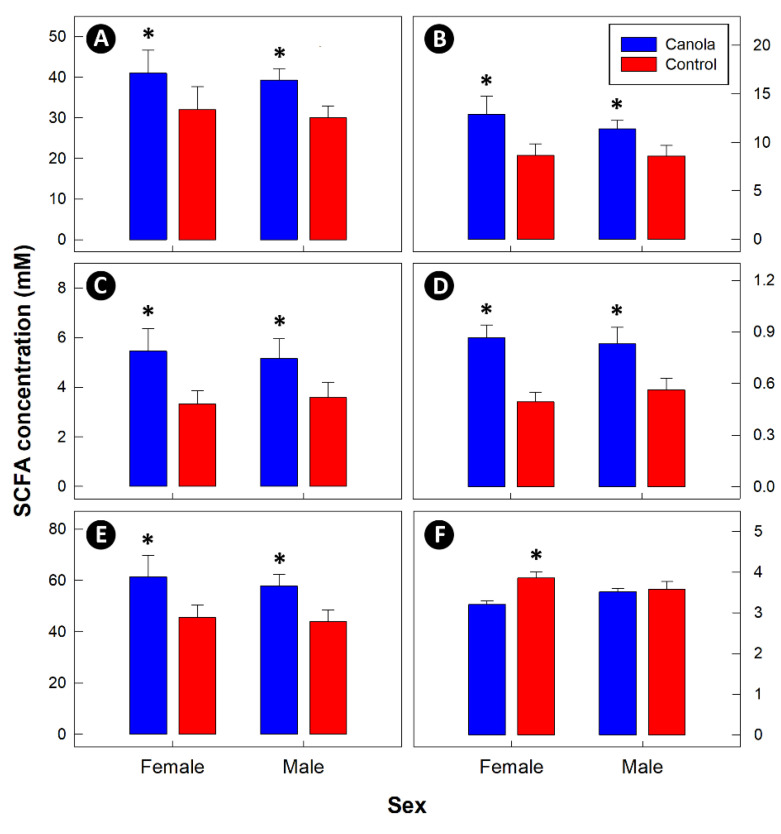
Short-chain fatty acid (SCFA) concentrations in the cecal digesta of 35-day-old broilers fed a diet supplemented with canola meal (20%) relative to a control diet as measured by gas chromatography. (**A**) Acetate. (**B**) Proprionate. (**C**) Butyrate. (**D**) Valerate. (**E**) Total SCFAs. (**F**) Ratio of two carbon to three carbon SCFAs. There was no difference (*p* ≥ 0.549) between females and males, and in both sexes higher (*p* ≤ 0.025) concentrations of SCFAs were observed in the cecal digesta of birds fed the diet with canola meal (indicated by asterisks).

Dietary fiber was higher in the CM diet (Table 2). There was no difference (*p* ≥ 0.549) in the cecal concentrations of SCFAs between males and females (Figure 5). However, concentrations of acetate (*p* = 0.025), propionate (*p* = 0.013), butyrate (*p* = 0.018), valerate (*p* < 0.001), and total SCFAs (*p* = 0.017) were higher in the ceca of birds fed the CM diet. In contrast, there was no difference (*p* ≥ 0.069) in concentrations of isobutyrate, isovalerate, and caproate between the two diet treatments. A higher (*p* = 0.043) ratio of two carbon to three carbon SCFAs was only observed in female birds fed the CM diet.

### 3.5. Metabolite Profiles in the Cecal Digesta, Pancreas, Liver, and Breast Muscle Were Altered in Broilers Fed the Canola Meal Diet

**Figure 6:**
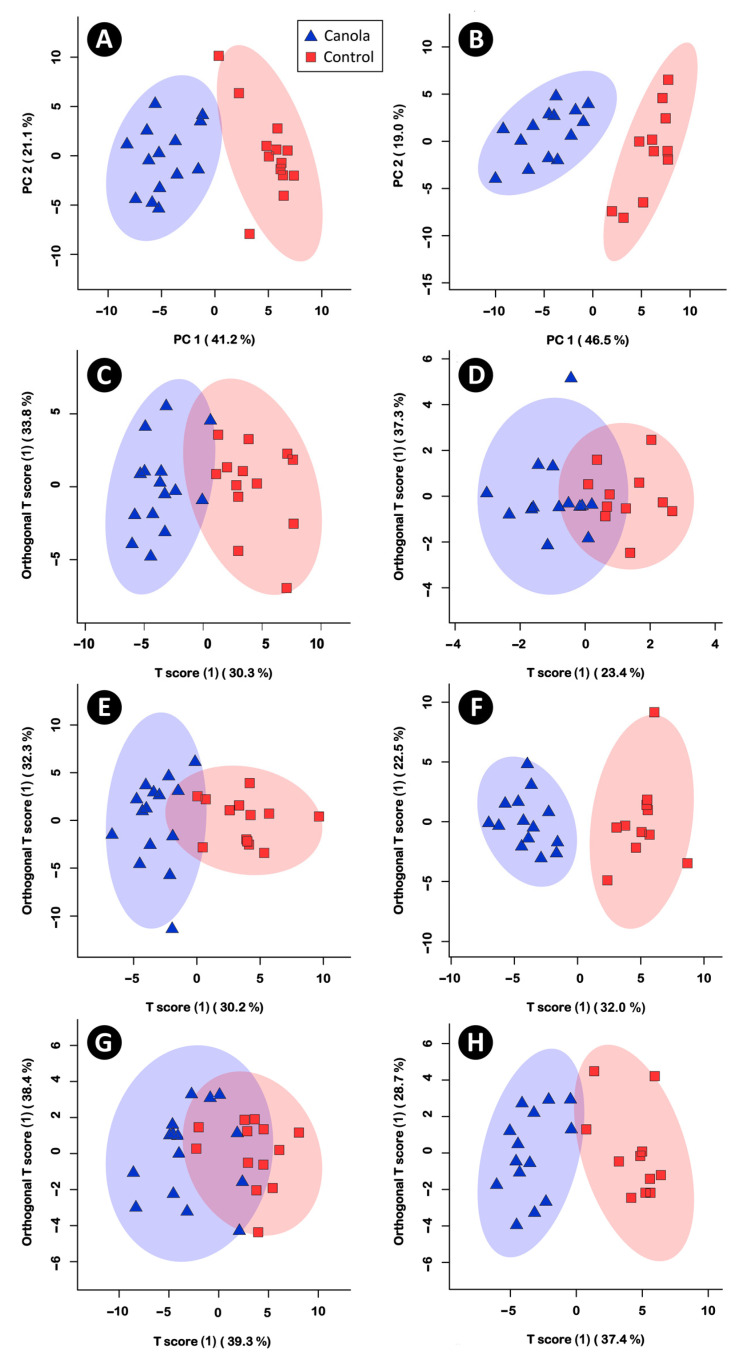
(**A**,**B**) Principle Component Analysis (PCA) showing unsupervised multivariate analysis of the cecal digesta metabolome among female (left) and male (right) from birds fed a diet supplemented with canola meal (20%) or a control diet. (**C**–**H**). Orthogonal Projections to Latent Structures Discriminant Analysis (OPLS-DA) showing supervised separation (*p* ≤ 0.014) for female (left) and male (right) birds fed the two diets. (**C**,**D**) Pancreas. (**E**,**F**) Liver. (**G**,**H**) Breast muscle. Each triangle or square represents one individual bird, and ellipsoids represent significant clustering by diet treatment at 95% confidence interval. For Figures **A**,**B**, the x-axis represents principle component 1, and the y-axis represents principle component 2. For Figures **C**–**H**, the x-axis represents the between-group variance (T-score), and the y-axis represents within-group variance (orthogonal T-score). Percent values in parenthesis show variances.

**Figure 7:**
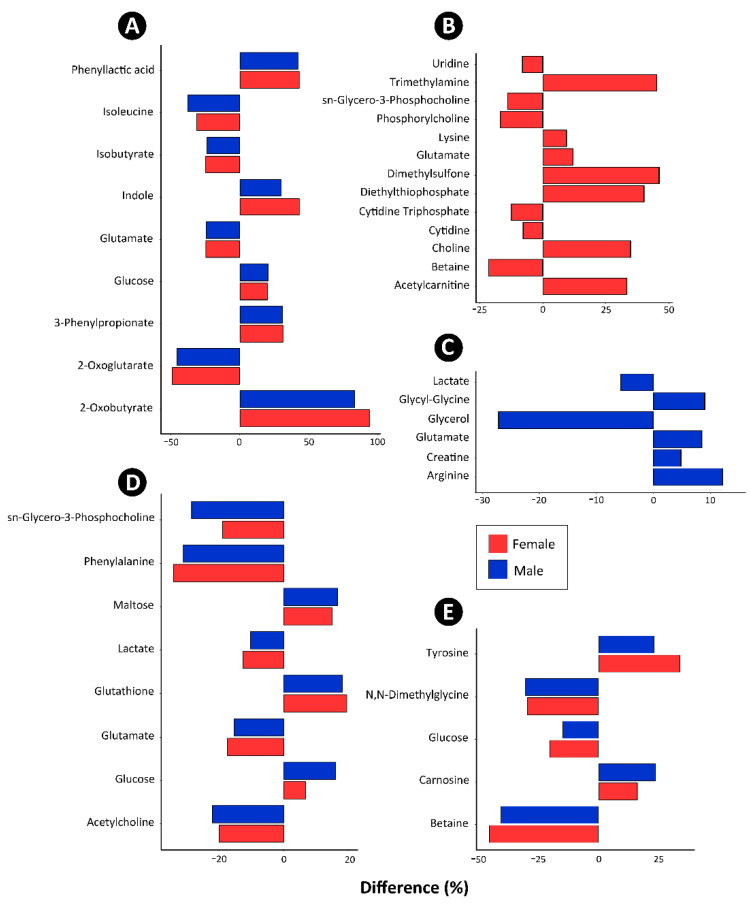
Average percent differences of individual metabolite quantities associated with female and male birds fed a diet supplemented with canola meal (20%) or a control diet as identified by a Mann-Whitney U test. All metabolites shown were significantly altered based on a p-value less than 0.05. (**A**) Cecal digesta. (**B**) Pancreas from female birds. (**C**) Pancreas from male birds (**D**) Liver. (**E**) Breast muscle. Negative values are less abundant, and positive values are more abundant in birds fed the canola meal diet.

The analysis of water-soluble metabolites in cecal digesta using NMR spectroscopy yielded 418 spectral bins. Unsupervised PCA indicated that the CM diet affected metabolite profiles in both female (177 significant bins) and male (195 significant bins) broilers (Figure 6A,B). Individual metabolites that were conspicuously lower in abundance (*p* ≤ 0.007) in both male and female birds fed the CM diet included isoleucine, isobutyrate, glutamate, and 2-oxoglutarate (Figure 7A). Whereas phenyllactic acid, indole, glucose, 3-phenylproprionate, and 2-oxobutyrate were more abundant. 

Five hundred and thirty-four, 350, and 428 spectral bins were obtained from pancreas, liver, and breast muscle tissue, respectively. Supervised OPLS-DA indicated that the CM diet affected the metabolite profiles of the pancreas as compared to the control diet in female (Figure 6C) and male (Figure 6D) broilers; in female chickens, 67 metabolite bins were affected (*p* < 0.001; Q^2^ = 0.63; R^2^ = 0.78), and in male chickens, nine bins were affected (*p* = 0.014; Q^2^ = 0.19; R^2^ = 0.62). In the liver of female chickens, 56 metabolite bins differed (*p* < 0.001; Q^2^ = 0.57; R^2^ =0.75) between birds that were fed the CM and control diets (Figure 6E), and in the liver of male chickens, 74 bins differed (*p* = 0.001; Q^2^ = 0.58; R^2^ = 0.89) between diets (Figure 6F). Similarly, the metabolite profiles of female (Figure 6G) and male (Figure 6H) broiler breast muscle differed between birds that were fed the CM and control diets. In this regard, 52 (*p* = 0.012; Q^2^ = 0.26; R^2^ = 0.51) and 50 (*p* < 0.001; Q^2^ = 0.64; R^2^ = 0.84) metabolite bins were altered between diets for female and male broilers, respectively. A number of specific metabolites were differentially abundant in the pancreas, liver, and breast muscle tissues of male and female chickens fed the two diets (Figure 7B–E). In the pancreas, the relative abundance of metabolites differed between female and male birds (Figure 7B,C); in both sexes, increased concentrations of glutamate were observed in birds that were fed the CM diet. In liver tissue, sn-glycero-3-phosphocholine, phenylalanine, lactate, glutamate, and acetylcholine were less abundant in female and male broilers, and maltose, glutathione, and glucose were more abundant in birds fed the CM diet (Figure 7D). Carnosine and tyrosine were more abundant, and N,N-Dimethylglycine, glucose, and betaine were less abundant in the breast muscle from broilers fed the CM diet (Figure 7E). 

## 4. Discussion

### 4.1. Inoculation of Birds with Cecal Digesta

The impacts of a diet that was supplemented with CM on the growth of broilers, cecal microbiota structure and function, and metabolome of pancreas, liver, and breast muscle was examined. The microbiota of the intestine is important for optimal bird health [30,31,32,33], and the ingestion of microorganisms present in the environment is a crucial strategy by which broiler chicks populate their gastrointestinal tract [34]. Moreover, it is likely that this microorganism acquisition strategy contributes to the variation in the structure of the microbiota that is commonly observed among individual birds within a flock. Birds were maintained in a sanitized facility, as one goal of the current study was to ascertain the impact of CM on the structure and function of the enteric microbiota. We observed that the diversity of the microbiota (Shannon’s index of ≈6.8) was comparative to that of ceca harvested from broilers in commercial production settings [35]. Moreover, the structure of the microbiota amongst broilers maintained in replicate housing enclosures was consistent, which indicated that the establishing a diverse cecal microbiota via the administration of bacteria from a donor bird was successful. It is noteworthy that others have inoculated neonatal chicks with digesta harvested from donor birds, which imparted long-term impacts on the behavior and physiology of birds [36].

### 4.2. Canola Meal Characteristics and Impacts on Bird Growth and Nutrition

CM and RM contain high amounts of protein [2], but both have been shown to be anti-nutritive in poultry and other livestock species [4,7]. Oil is extracted from canola seeds by a variety of methods, and the extraction method used has been shown to influence the chemical and nutritive characteristics of CM, primarily as a result of heat treatment [5,6]. Notably, the expeller-pressed CM used in the current study, which involves the physical separation of oil from meal without heat can contain 8–12% oil in comparison to solvent-extracted CM that typically contain less than 1% oil [37]. This is a substantially higher oil content than soybean meal, which increases the relative energy content that is present in CM [38]. However, given the higher fiber content, CM comparatively contains a lower metabolizable energy level than other protein sources, such as soybean meal in poultry diets [39], which dilutes the energy content [40]. We formulated the CM and soybean meal control diets to be equivalent in metabolizable energy, and despite the presence of known anti-nutritional materials in CM, a detrimental impact of CM on feed consumption and/or growth of broilers was not observed over a 35-day period, which is a common termination point for broilers in Alberta [41]. Others have also observed no impacts on feed intake in broilers that were maintained on diets supplemented with CM up to a concentration of 20–30% [42,43,44]. To prevent impacts of CM on diet intake and performance, proper formulation, and in particular, the concentrations of ileal digestible amino acids, is important [45]. In this regard, the CM diet in the current study was specifically formulated to be equivalent to the soybean diet with respect to the digestible amino acid content.

### 4.3. Impacts of Canola Meal on the Cecal Microbiota

The impact of CM and RM on the intestinal microbiota structure and function has not been extensively examined in chickens. We observed that the CM diet did not affect the diversity or evenness of the cecal microbiota, and there were no differences in female versus male broilers. Bacterial fermentation primarily takes place in the ceca of chickens [46], and a number of metabolites are produced via the fermentation of dietary components (e.g., proteins and polysaccharides); SCFAs are considered to be of particular importance in animal health. The primary SCFAs produced in the intestine of animals as end products of the fermentation of complex organic molecules are acetate, propionate, and butyrate [47,48]. Other SCFAs, such as valerate, are generated in lesser quantities. Concentrations of acetate, butyrate, propionate, and valerate were significantly elevated in the cecal digesta of birds fed the CM diet, but there was no difference between the female and male birds. A salient difference between the CM and soybean meal diets evaluated in the current study was the substantially higher concentration of fiber in the former (i.e., crude, acid detergent, and neutral detergent fiber). The fermentation of non-starch polysaccharides in RM within the ceca of chickens facilitated by colon reflux has been reported previously, albeit at relatively low levels [49]. Thus, the elevated concentrations of SCFAs that we observed in the ceca of birds that were fed the CM diet was anticipated. Although the optimal feed efficiency in production chickens does not rely on bacterial fermentation within ceca [50], the fermentation of complex molecules has been shown to impart positive non-nutritional impacts on the health of animals [51]. Although SCFAs can have a positive impact on bird health, they may also adversely affect animals in various ways, including the alteration of gut-brain axis signaling and brain physiology [52,53]. Although a relationship between the increased levels of SCFAs, and feed efficiency or overall health of birds fed the CM diet was not observed, it is possible that the elevated levels of SCFAs observed within the cecum may have imparted cryptic positive health benefits.

The cecal microbiota was observed to be dominated by *Firmicutes* and *Bacteroidetes*, and *Actinobacteria*, *Proteobacteria*, *Synergistetes*, *Spirochaetes*, and *Verrucomicrobia*, as has been observed previously [54]. Consistent with the observed increases in SCFA concentrations, the addition of CM to the diet affected the structure of the cecal microbiota (i.e., β-diversity). In this regard, the abundance of bacterial families containing members that are able to ferment dietary fiber increased. For example, bacteria in the *Methanobacteriaceae*, *Atopobiaceae*, *Prevotellaceae*, *Clostridiales* Family XIII, *Peptostreptococcaceae*, and *Succinivibrionaceae* were more abundant in the ceca of birds that were fed the diet supplemented with CM. 

### 4.4. Impacts of Canola Meal on the Cecal Metabolome

Metabolomics was applied to obtain a direct functional readout of the physiological state of microbial activity [55] within the digesta of chickens that were fed the CM diet. Consistent with our findings of differential SCFA concentrations, the metabolome of cecal digesta was conspicuously affected in birds ingesting the CM diet. In this regard, >36 metabolites were altered, and nine were associated with SCFA metabolism. More specifically, 2-oxobutyrate was found to be substantially more abundant in the digesta of CM treatment birds; this metabolite is involved in many pathways, including the degradation of methionine and threonine into propionate [56]. A lower concentration of 2-oxoglutarate was observed in birds that were maintained on the CM diet; this metabolite has emerged as a master regulator, and the levels of this metabolite have been shown to coordinate multiple carbon-dedicated pathways, such as fatty acid production [57]. Phenyllactic acid, which is a derivative of propionate, was also more abundant in digesta of birds fed the CM diet, and it is a product of phenylalanine catabolism found in several enteric bacteria [58]. Higher concentrations of 3-phenylpropionate were observed in the digesta of CM treatment birds, a derivative of propionate that is associated with phenylalanine metabolism [59]. Bacteria within the *Peptostreptococcaceae* are able to use glutamate to produce acetate and butyrate [60]. The lower concentrations of glutamate observed in the cecal digesta of birds fed the CM diet is consistent with the utilization of glutamate by this group of bacteria. The elevated quantities of glucose observed in the cecal digesta of birds provided the CM diet suggests that either assimilation was reduced or that the increased protein in the CM diet increased the conversion of protein by bacteria into glucose for energy use. *Prevotella* spp. are known to produce propionate via the fermentation of aromatic amino acids, and tyrosine to produce indole as a by-product [61]. In this regard, the increased abundance of indole in the digesta of birds that were fed the CM diet is consistent with the higher total protein content of the CM diet. Isobutyrate was lower in the digesta of birds fed the CM diet; this metabolite functions as a carbon source for energy in colonocytes, and bacterial taxa (e.g., some *Firmicutes* and methanogens) are able to convert isobutryate to butyrate [62]; thus, the higher amounts of butyrate that were observed are consistent with the reduced abundance of isobutyrate detected in digesta as a result of utilization by enteric bacteria.

### 4.5. Impacts of Canola Meal on the Metabolome of the Pancreas

The pancreas has both exocrine and endocrine functions that are crucial in the digestion [63] and regulation of blood sugar levels [64]. Sexually dimorphism in pancreatic function occurs in animals, including chickens [65,66], and we observed that the relative quantities of metabolites differed between male and female birds. The most pronounced effect of the CM diet on the metabolome occurred in female birds (13 metabolites were affected). Some of the metabolites that were differentially abundant have been previously linked to the early stages of pancreatitis or pancreatitis-like effects in other animals. An increase in lysine, a basic amino acid, was observed in female birds provided the CM diet. This amino acid has also been shown to selectively impair the function of ATP synthase of pancreatic mitochondria at high concentrations, causing oxidative stress, followed by inflammation [67]. Increased choline, along with decreased sn-glycero-3-phosphocholine, phosphorylcholine, betaine, and glycine levels, have been previously identified as a pattern of early stages of pancreatitis in swine [68]. In contrast, other metabolites that differed between female birds maintained on the two diet treatments have been linked to a protective effect. Betaine, which can be synthesized from choline, along with acetylcarnitine and dimethylsulfone, have been shown have antioxidant effects that would help counter pancreatitis-like effects [68]. Changes in the relative concentrations of these protective metabolites could be a response to the increased concentrations of metabolites that are known to cause damage to the pancreas. 

In male birds, five metabolites that were involved in energy production were affected by the diet. Lactate is an important energy metabolite that is a product of glycolysis, an energy producing pathway, and it can also be re-used as an energy source by being re-converted to glucose via gluconeogenesis. The fact that both lactate and glycerol, which are also involved in gluconeogenesis, were less abundant in the pancreases of chickens fed the CM diet suggests that rates of gluconeogenesis were increased as the birds produced more glucose for energy. Creatine was observed to be more abundant in male birds that were fed the CM diet. Creatine is produced from arginine, glycine, and methionine in the pancreas [69], and it has also been observed to act on pancreatic beta cells to increase intracellular ATP and enhance insulin secretion in the presence of glucose [70]. Arginine is a metabolite that is safe at low concentrations, but can be harmful to pancreatic tissue at higher concentrations. Because of this, it is often used to induce pancreatitis in experimental animals [71]; it does this by damaging mitochondria, which decreases the energy potential of the cells and causes oxidative stress [67]. Glutamate was the only significantly altered metabolite that was common to the male and female pancreatic tissue; however, it is unclear why glutamate increased in chickens that were provided the CM diet. Although the CM diet did not affect feed intake and bird growth, the changes that were observed in the relative concentration of several metabolites in the pancreases of female and male broilers fed CM indicate the possibility of increased risk for pancreatitis and oxidative damage. Thus, pancreas health should be further investigated and monitored when studying the effects of CM and other diet supplements on chickens.

### 4.6. Impacts of Canola Meal on the Metabolome of the Liver

The liver is crucial for nutrition in chickens, including bile formation, the metabolism of carbohydrates, lipids, and protein, and storage of glycogen, fat, and fat-soluble vitamins. Moreover, the liver plays an essential role in the detoxification of ingested substances, and evidence of pathologic changes to liver that were linked to glucosinolates in RM have been observed previously [72]. We observed differences in the liver metabolome of broilers that were fed the CM diet when compared to chickens maintained on the control diet. Salient changes included increased abundance of maltose, glutathione, and glucose, and a decreased abundance of sn-glycero-3-phosphocholine, phenylalanine, lactate, glutamate, and acetylcholine. Hepatic gluconeogenesis involves the conversion of amino acids, lactate, and pyruvate into glucose [73], and the lower abundance of lactate and increased abundance of glucose observed in the livers of broilers fed the CM diet is consistent with the increased use of energy reserves in these birds.

Protein metabolism in the liver is linked to amino acid synthesis, including glutathione metabolism. Glutathione is upregulated, and it is an antioxidant that is used to scavenge free radical and reactive oxygen species (ROS). Moreover, glutathione is synthesized from glutamate, cysteine, and glycine. Thus, the reduced abundance of glutamate and corresponding decrease in abundance of glutathione suggests that the livers of birds fed the CM diet is either working more efficiently to protect the body from ROS, or it is having to work harder as a result of more ROS being present. The liver is involved in lipid metabolism, including glycerophospholipid metabolism, as indicated previously. The increased abundance of sn-glycero-3-phosphocholine and reduced abundance of acetylcholine in birds fed the CM diet are consistent with increased glycerolphospholipid metabolism; sn-glycero-3-phosphocholine is an upstream metabolite that is converted into choline and then to acetylcholine. Acetylcholine receptors are expressed throughout the liver and are connected to parasympathetic nerve fibers [74], and nicotinic acetylcholine receptors are important to the initial phase of liver regeneration, as well supporting the survival of hepatic cells [74]. The decreased abundance of both sn-glycero-3-phosphocholine and acetylcholine in birds that were fed the CM diet suggests that there may be either less of a need for production of cell membrane components in hepatic cells, or an inhibited ability to produce those structural membrane components. The substantive decrease in phenylalanine that was observed in birds provided the CM diet may be associated with a reduced ability to correctly respond to stimuli; phenylalanine is a precursor for tyrosine, which is then used for dopamine, epinephrine, and norepinephrine synthesis [75]. Similarly to the pancreas, an analysis of the metabolome of liver revealed changes in metabolism in broilers that were fed the diet supplemented with CM that could indicate an impairment of bird health.

### 4.7. Impacts of Canola Meal on the Metabolome of Breast Muscle

Although male broiler breast muscle tends to be larger and have higher nutrient requirements to reach larger mass [76], we did not observe any sex specific differences in the metabolome of breast muscles; however, the metabolite profiles of breast muscle did differ between the two diet treatments. Many of the differentially abundant metabolites were connected to the muscular energy levels. In this regard, the breast muscle of chickens that were fed the CM diet had a lower relative concentration of glucose. Glucose is oxidized as a source of ATP in muscle cells, which equates to less energy potential being stored in the muscle. Betaine also affects the energy levels in muscle cells through its actions as an osmolyte; it can lower the required activity of sodium-potassium ATPases to reduce the energy expenditure of muscle cells [77]. Broilers have also previously been shown to use N,N-dimethylglycine to help compensate for lower energy diets [78]. Whether the metabolomic changes observed in chickens fed the CM diet are due to the diet providing higher energy, or a result of the chickens working metabolically harder to produce energy due to a deficit is unclear and warrants further investigation. Some of the metabolites that were altered in the breast muscle have also been associated with a protective role against oxidative stress. For example, N,N-dimethylglycine, carnosine, and betaine have antioxidant properties that allow for them to counteract the effects of ROS [77,78,79].

## 5. Conclusions

The inclusion of expeller-pressed CM in a broiler diet did not affect the feed consumption or weight gain of broiler chickens that were housed in a controlled setting. However, the CM diet influenced the structure of the cecal microbiota selecting for bacterial taxa involved in fiber catabolism, which was associated with the increased production of SCFAs. Even though the energy intake was equivalent for the two diet treatments, the metabolic profiles of cecal digesta, pancreas, liver, and breast muscle of birds fed the CM diet significantly differed from birds that were maintained on the control diet. Some of the altered metabolites were linked to the action of enteric bacteria. Others were linked to energy production, protection against oxidative stress, and changes to pathways of amino acid and glycerophospholipid metabolism. Importantly, the study demonstrated the power of using metabolomics to study diet impacts in poultry science, and to identify biomarkers of bird health that may be used as the baselines for evaluating chicken responses to diets, including those that are associated with potential adverse impacts of CM and other feed supplements. The combined metabolomic changes that were observed in the current study indicate that further investigation into the potential health effects of CM supplementation of diets on female and male broiler chickens in production settings is warranted.

## Figures and Tables

**Table 1:** Composition of the control and canola meal broiler diets.

Ingredient	Control (%)			Canola Meal (%)		
	Starter	Grower	Finisher	Starter	Grower	Finisher
Corn	49.53	54.68	59.84	38.02	44.75	48.35
Canola meal	‒	‒	‒	20.00	20.00	20.00
Soybean meal	43.06	37.31	31.57	34.60	25.40	23.11
Canola oil	2.39	3.26	4.12	2.88	5.52	4.61
Salt	0.51	0.52	0.52	0.49	0.51	0.50
Limestone	1.52	1.41	1.31	1.35	1.18	1.13
Dicalcium phosphate	1.26	1.09	0.92	1.12	1.08	0.78
Magnesium oxide	0.10	0.15	0.17	0.02	0.06	0.09
L-lysine HCl	0.11	0.12	0.13	0.08	0.14	0.10
D,L-methionine	0.37	0.33	0.31	0.30	0.24	0.24
L-threonine	0.15	0.13	0.11	0.14	0.12	0.09
Vitamin premix	0.50	0.50	0.50	0.50	0.50	0.50
Choline premix	0.50	0.50	0.50	0.50	0.50	0.50

**Table 2:** Nutrient analysis of the control and canola meal broiler diets.

Ingredient	Control			Canola Meal		
	Starter	Grower	Finisher	Starter	Grower	Finisher
Metabolizable energy (Mcal/kg)	3.00	3.10	3.20	3.00	3.10	3.20
Dry matter (%)	90.49	90.73	90.53	91.11	91.49	90.70
Crude fiber (%)	3.81	3.57	3.33	4.46	4.97	3.97
Acid detergent fiber (%)	5.15	4.86	4.56	7.29	7.45	6.70
Neutral detergent fiber (%)	8.53	8.53	8.53	10.70	12.49	10.69
Total protein (%)	24.20	21.90	19.60	26.30	23.26	21.70
Digestible protein (%)	20.96	19.01	17.06	21.09	18.65	17.18
Fat (%)	4.41	5.27	6.13	6.89	7.76	8.60
Calcium (%)	1.01	0.91	0.82	1.01	0.91	0.82
Available phosphorus (%)	0.50	0.46	0.41	0.50	0.46	0.40
Magnesium (%)	0.25	0.26	0.26	0.25	0.26	0.26
Sodium (%)	0.21	0.21	0.21	0.21	0.21	0.21
Linoleic acid (%)	1.62	1.92	2.22	1.97	2.20	2.58
Digestible arginine (%)	1.48	1.32	1.16	1.49	1.29	1.17
Digestible histidine (%)	0.56	0.51	0.46	0.58	0.53	0.47
Digestible isoleucine (%)	0.95	0.85	0.76	0.93	0.82	0.74
Digestible leucine (%)	1.75	1.61	1.48	1.71	1.55	1.45
Digestible lysine (%)	1.34	1.21	1.07	1.34	1.21	1.07
Digestible methionine (%)	0.69	0.63	0.58	0.66	0.57	0.54
Digestible cysteine (%)	0.31	0.28	0.26	0.34	0.34	0.30
Digestible phenylalanine (%)	1.07	0.97	0.86	1.04	0.92	0.83
Digestible threonine (%)	0.90	0.81	0.71	0.90	0.81	0.71
Digestible tryptophan (%)	0.29	0.25	0.22	0.29	0.26	0.22
Digestible valine (%)	1.01	0.91	0.82	1.01	0.91	0.82

## Data Availability

The data presented in this study are available on request from the corresponding authors. The raw sequencing reads of bacterial community DNA have been submitted to the Sequencing Read Archive of NCBI under BioProject accession number PRJNA702534.

## Supplementary Materials

The following are available online at https://www.mdpi.com/2076-2615/11/2/577/s1, Figure S1. Rarefaction curves of bacterial Amplicon Sequence variants (ASVs) in the cecal digesta of 35-day-old broilers fed a diet supplemented with canola meal (20%) relative to a control diet. Figure S2. Diversity and evenness of bacterial communities in the cecal digesta of 35-day-old female and male broilers fed a diet supplemented with canola meal (20%) or a control diet.
