# Supplementary material for: Expeller-Pressed Canola (Brassica napus) Meal Modulates the Structure and Function of the Cecal Microbiota, and Alters the Metabolome of the Pancreas, Liver, and Breast Muscle of Broiler Chickens
Source: Animals (Basel). 2021 Feb 23;11(2):577. doi: 10.3390/ani11020577 (PMC7926547; doi:10.3390/ani11020577)
Supplement: Supplementary file 1 [file animals-11-00577-s001.pdf]

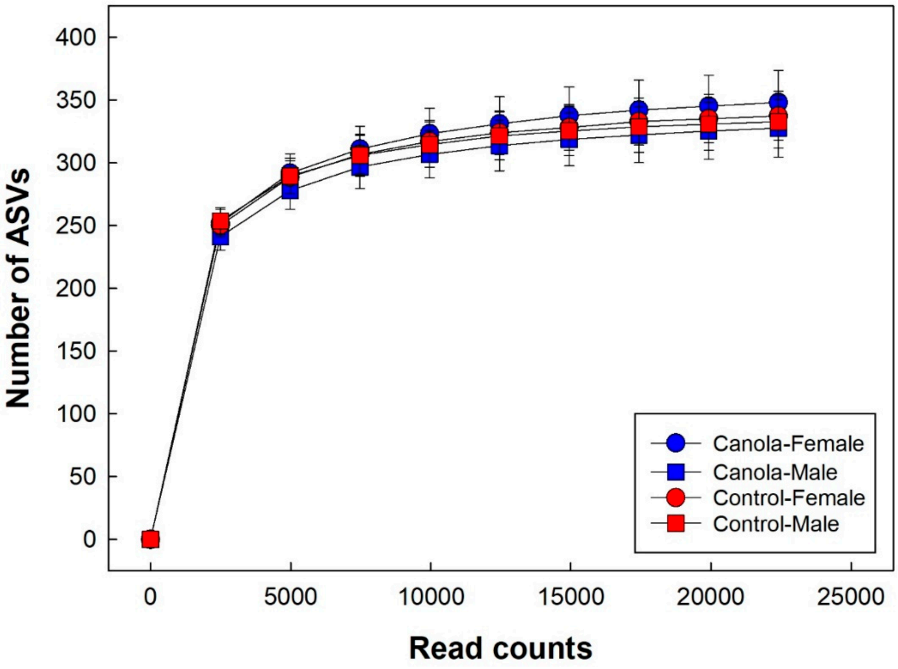

Figure S1. Rarefaction curves of bacterial Amplicon Sequence variants (ASVs) in the cecal digesta of 35-day-old broilers fed a diet supplemented with canola meal (20%) relative to a control diet.

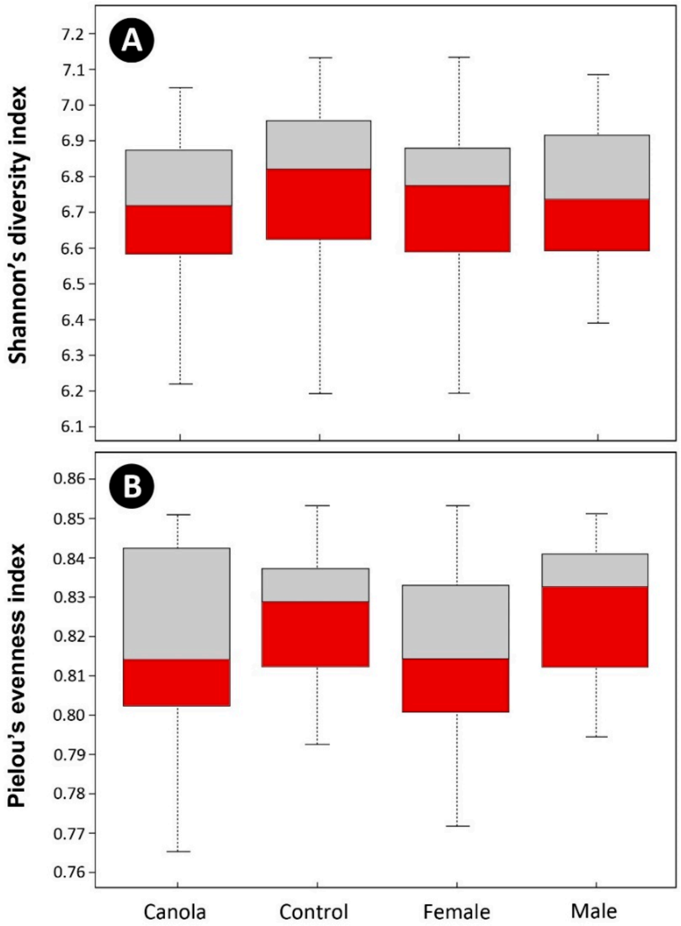

Figure S2. Diversity and evenness of bacterial communities in the cecal digesta of 35-day-old female and male broilers fed a diet supplemented with canola meal (20%) or a control diet. (A) Shannon's α diversity. (B) Pielou's evenness index. The center lines in the box plot represent the median value, the size of the box plot represents the distribution within a confidence of 95%, and the dotted vertical lines associated with the box plot represent the total spread of data. There was no difference ($P \geq 0.319$) in alpha diversity or evenness between the two diet treatments or broiler genders.
